# Winter Bathing: An Ice-Cold Strategy for Improving Quality of Life for People with Attention Deficit Hyperactivity Disorder

**DOI:** 10.3390/healthcare14060752

**Published:** 2026-03-17

**Authors:** Troels Holm Nielsen, Nikolai Winkler Karlsen, Lucas Tuan Minh Hoang, Jesper Dahlgaard, Elisabeth Bomholt Østergaard

**Affiliations:** 1 Department of Physiotherapy, VIA University College, 8200 Aarhus, Denmark; troelshn@hotmail.com (T.H.N.); nikolai.wk@hotmail.com (N.W.K.); tuan.minh.hoang@gmail.com (L.T.M.H.); 2 Research Programme for Mental Health, Research Centre for Prevention and Rehabilitation, VIA University College, 8200 Aarhus, Denmark; jesd@via.dk

**Keywords:** winter bathing, winter swimming, attention deficit hyperactivity disorder (ADHD), blue nature, blue space, ice-cold strategy, quality of life, mental well-being, mental peace, anchor to the present moment

## Abstract

**Background**: Attention deficit hyperactivity disorder (ADHD) is a neurodevelopmental condition with increasing prevalence of adult diagnoses in Denmark. ADHD is characterized by inattention, hyperactivity, and impulsiveness. Conventional treatment is primarily pharmacological. Many adults with ADHD face challenges in maintaining structure in daily life and have an increased risk of developing stress, anxiety, and depression. Winter bathing is gaining popularity and is associated with improvements in mood, sleep quality, and the ability to handle stress. **Objectives**: The aim of this study was to explore how winter bathing was experienced to affect the mental well-being of five adults diagnosed with ADHD. **Methods**: A qualitative research design was used, with participant observation and semi-structured interviews with five participants in April 2025. Additional data were obtained through interviews with a psychologist and an ADHD mentor. Empirical data were thematically analysed, followed by theoretical analysis. **Results**: Six themes were revealed: Mental calmness: peace of mind and relief from racing thoughts; Bodily awareness and connection to the body; Joy: sustained positivity; Nature: essential for motivation and general mind–body calmness; Coping with and managing everyday life better; and Winter bathing as a supplement or alternative to medication for anxiety, depression and ADHD. **Conclusions**: Five adults with ADHD experienced winter bathing as a meaningful and motivating non-pharmacological intervention that strengthened their mental well-being and quality of life. Winter bathing promoted general mind–body calmness, sustained joy, bodily connection, and mental coping, and was used as a supplement or alternative to medication. Nature emerged as a key motivational factor enhancing presence and well-being.

## 1. Introduction

In recent years, Denmark has experienced a significant increase in the number of adults diagnosed with attention deficit hyperactivity disorder (ADHD) [1]. Data from the Danish Health Data Authority indicate a steady increase in the use of ADHD medication, particularly among adults. ADHD medication refers to pharmacological treatments approved for ADHD, primarily divided into stimulant medications (methylphenidate, dexamfetamine, lisdexamfetamine) and non-stimulant medications (atomoxetine and guanfacine).

The most notable rise is observed in the 25-to-44 age group, where the number of individuals receiving ADHD medication has more than tripled over the past decade, reaching approximately 42,500 in 2023, corresponding to 2% of the Danish population [1,2].

ADHD is a neurodevelopmental condition, primarily caused by hereditary factors, which is behaviourally manifested in early childhood and persists into adulthood [3]. The condition is characterised by three core symptoms, which vary from person to person: inattention, hyperactivity, and impulsivity. Inattention involves difficulties maintaining focus, organising tasks, and remembering appointments. Hyperactivity can manifest as physical restlessness and a constant need for movement, while impulsivity refers to rash, unconsidered actions, impatience, and challenges with emotional regulation [3]. Not all individuals with ADHD exhibit all symptoms. A subtype, ADD (attention deficit disorder), is characterised primarily by attention difficulties without pronounced hyperactivity. In adulthood, symptoms such as concentration problems, restlessness, and, to some extent, impulsivity tend to dominate in comparison to hyperactivity, which is more prominent in childhood.

Many adults with ADHD experience considerable difficulties in creating structure and maintaining an overview in daily life, which can make it challenging to retain employment [1]. Furthermore, there is an elevated risk of impulsive behaviour, challenges with completion of education and adherence to employment, substance abuse, and the development of comorbid conditions such as stress, anxiety, and depression [4,5].

People with ADHD are also at increased risk of lower educational attainment, involvement in criminal activity and unemployment [1]. These factors pose both an individual and a societal burden, including increased expenditures on healthcare and social services. A Danish study from 2020 indicates that the average annual societal cost of a person with ADHD is DKK 120,000, corresponding to about EUR 16,000. When transfer payments are included, this figure increases to just over DKK 170,000, corresponding to about EUR 23,000 [6].

The conventional treatment is primarily pharmacological, involving both stimulant and non-stimulant medications to manage symptoms. However, pharmacological treatment can cause side effects in some individuals, such as increased heart rate and blood pressure, loss of appetite, and sleep disturbances [7], and it is also associated with significant public costs [6,7], highlighting the growing need for alternative or complementary treatment approaches [4,5,8]. For adults with ADHD who have difficulties taking medication every day, whose medication is not working or cannot be tolerated, and for those who have made an informed choice not to receive pharmacological treatment, non-pharmacological treatments may be relevant [5]. The Danish Health Authority also recommends non-pharmacological approaches, such as psychoeducation [5], and when symptoms of anxiety or depression are also present, cognitive behavioural therapy may be employed [7]. In response to the increasing number of adults diagnosed with ADHD, the Danish Health Authority has developed new national clinical recommendations for the assessment and treatment of adults with ADHD (5). These clinical recommendations recommend (1) considering non-pharmacological treatment, specifically cognitive behavioral therapy, in addition to medication for adults with ADHD, and (2) considering such treatment for adults with ADHD who are not receiving medication.

When functional capacity or quality of life is affected, psychological interventions such as cognitive behavioral therapy, dialectical behavior therapy, a combination of the two, mindfulness, mindfulness-based cognitive therapy, cognitive training, or psychosocial interventions may be relevant in the treatment of adults with ADHD [5].

However, the data and evidence regarding their efficacy are still inconclusive [9].

The National Institute for Health and Care Excellence highlights that non-pharmacological treatments may have potential as adjuncts to pharmacological interventions, but that the evidence base remains limited [10]. A systematic review and meta-analysis [11] also indicates that although pharmacological treatment is effective, evidence for non-pharmacological interventions in adults with ADHD remains scarce, reinforcing the need for further research and development of complementary treatment options that may support mental well-being among those who do not fully benefit from conventional treatment or who wish to avoid potential side effects or dependence on pharmacological medication. Thus, complementary treatment approaches for ADHD may be explored, ideally considering both biological and psychological mechanisms involved in its manifestation.

Research suggests that ADHD is associated with alterations in brain neurotransmitters, particularly dopamine and noradrenaline, which contribute to difficulties in regulating attention and impulse control [3]. Winter bathing, with exposure to cold water, activates several psychoneuroimmunological and physiological mechanisms, including an acute stress response involving increased secretion of adrenaline and noradrenaline, followed by activation of the parasympathetic nervous system. This may contribute to a sense of mental calm and well-being afterwards [12]. Research [13,14] suggests that winter bathing can improve circulation and fat burning, reduce inflammation, strengthen the immune system, and enhance the body’s general resilience to stressors. A recent randomised study from 2023 showed that a single session of exposure to cold water reduces cortisol levels and has psychological implications with reduced negative affect in adults [15]. A qualitative study from 2024 [16] showed that regular winter bathing one to three times a week, for a minimum of one year and up to three years, had a positive impact on the participants’ mental health and well-being, including their comprehensibility, manageability, and meaningfulness, and the participants felt that mental peace and an increased sense of coherence were transferred to other situations in their daily lives and to life in general. Research also indicates that winter bathing and exposure to blue space surroundings have a positive impact on mental health parameters such as mood, sleep quality, and the ability to cope with stress [17,18,19,20]. However, in exploring winter bathing as a complementary treatment for ADHD, there is an empirical gap and a need for further research, especially in qualitative studies, to gain insight into experiences with winter bathing among people with ADHD, including its long-term impact on their mental health [14,16,19,21].

With its 8750 km coastline and a maximum distance of 50 km to open water from any point in the country, Denmark offers unique geographic conditions for access to lakes, fjords, and the sea [22]. In recent years, winter bathing has experienced a marked increase in popularity in Denmark, growing from 25,000 to 75,000 members over a decade, with long waiting lists in many winter bathing clubs [23]. Since a large part of the population has access to coastal waters and lakes, it may be relevant and feasible to explore how winter bathing is experienced in the context of ADHD and mental well-being among individuals living with this condition and whether winter bathing may contribute to an improved quality of life.

We examined the hypothesis that winter bathing may constitute a beneficial supplement or alternative to conventional treatment of individuals with ADHD in Denmark. The hypothesis is motivated by (1) the presence of potentially relevant biological and psychological mechanisms mediated by winter bathing for coping with ADHD, and (2) the wide accessibility of winter bathing in Denmark. More specifically, the aim of this study was to explore how winter bathing was experienced to affect the mental well-being of five adults diagnosed with ADHD.

### 1.1. Definitions

Winter bathing refers to the practice of regularly bathing in cold water in nature from autumn to spring. The use of a sauna is optional [18,24].

A winter bathing club is defined as a physical location where individuals can regularly bathe and participate in a social community with other winter bathers. Facilities typically include changing rooms, a sauna, and access, such as a staircase leading into the water.

Cold-water exposure refers to the body being exposed to cold water in a tub or similar setting and does not necessarily take place in open water such as rivers, lakes, the sea, or fjords.

**Figure 1:**
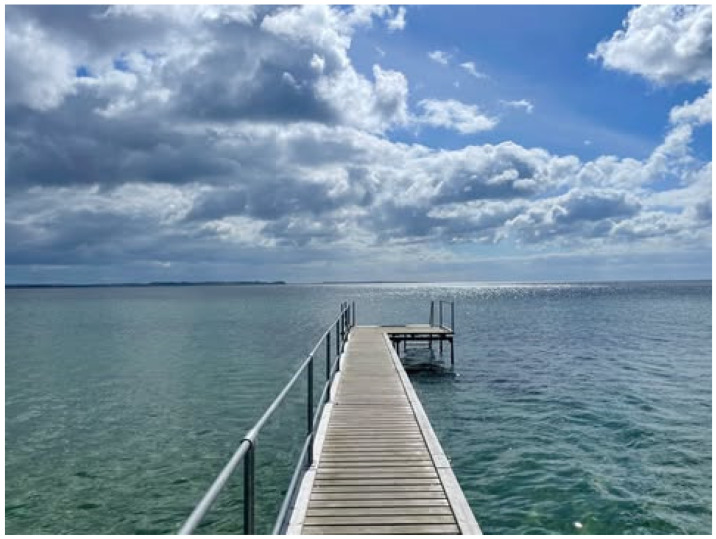
Winter bathing in blue nature. Photo: Lucas Tuan Minh Hoang.

Nature is understood as the outdoor environment in which winter bathing occurs, including the sea, fjords, rivers, or lakes, as well as forests and open skies. This definition is based on the WHO’s understanding of green spaces, which encompasses areas with natural vegetation and blue nature [25,26]. An example of blue nature is illustrated in Figure 1.

Mental well-being is used throughout the project and is defined by the WHO as a positive state that can be experienced by both individuals and communities. The concept encompasses quality of life and the ability to contribute to the world with a sense of meaning and purpose [27].

### 1.2. Theoretical Perspectives

Our theoretical perspectives were based on the phenomenology of perception by Maurice Merleau-Ponty [28,29], the concept of Sense of Coherence by Aaron Antonovsky [30], self-efficacy by Albert Bandura [31], Self-Determination Theory by Deci and Ryan [32,33,34,35], biophilia by Edward O. Wilson and Stephen R. Kellert [36,37], and the Attention Restoration Theory (ART) by Kaplan and Kaplan [38].

The phenomenology of perception, as developed by Maurice Merleau-Ponty, does not view the body merely as a biological object, but rather as the primary foundation for human experience of the world [28,29]. In contrast to traditional dualistic conceptions, separating body and mind, Merleau-Ponty argues that the human body and mind are inextricably linked and must be understood as an integrated whole. He describes the body as a “lived body”—something we are, not merely something we have [28,29]. Thus, we perceive, experience, and understand the world through the body, not primarily through thoughts or conscious cognition, as was previously assumed. In practice, Merleau-Ponty describes the pre-reflective body, which acts and senses without requiring conscious deliberation. This encompasses the understanding that the body possesses a unique form of knowledge, and, in conjunction with reflective awareness, an intentionality that is always directed toward something. Being present in the body enhances connection to the world, to the present moment, fostering presence and awareness [28]. In this way, the body plays an active role in how we make sense of the world and develop as human beings.

Aaron Antonovsky developed the theory of Sense of Coherence (SOC) and introduced the concept of salutogenesis [30]. SOC describes the relationship between stressors, coping, and health, positing that a person’s experience of coherence and meaning plays a crucial role in their ability to handle adversity and manage stress. The theory comprises three components: comprehensibility, manageability, and meaningfulness as a strategy to maintain health. Comprehensibility refers to the individual’s capacity to structure and make sense of internal and external stimuli. Manageability presupposes that adequate resources are available to meet these demands. Most important is meaningfulness, which emerges when challenges are perceived as worthwhile engaging [30].

Albert Bandura formulated the Social Cognitive Theory (SCT), in which one of the key concepts, reciprocal determinism, describes the dynamic interplay between the individual and their environment [31]. This interaction involves personal factors, behaviour, and external surroundings, all of which influence one another. Personal factors encompass cognitive, affective, and biological aspects. Behaviour is shaped by the interaction between external stimuli, internal processes, and responses from the environment. External surroundings include both the physical environment and other people.

Self-Determination Theory (SDT), developed by Edward L. Deci and Richard M. Ryan [32,33,34,35], focuses on the psychological prerequisites that foster human motivation. The theory highlights that the most enduring and deeply rooted form of motivation is intrinsic motivation, which occurs when individuals engage in an activity because it is inherently meaningful and satisfying. For intrinsic motivation to arise, three basic psychological needs must be fulfilled: competence, autonomy, and relatedness [35]. Competence refers to the feeling of being capable of mastering tasks and facing challenges. Autonomy concerns the experience of self-determination and having control over one’s actions and decisions. Relatedness refers to feeling connected to others, being acknowledged, and being part of a community. When these psychological needs are met, individuals are more likely to thrive and to act out of intrinsic motivation.

The Biophilia Hypothesis, developed by Edward O. Wilson [36], posits that humans have an innate affinity for nature, shaped by evolutionary conditions essential to human survival over millions of years. According to Wilson, human well-being is dependent on contact with natural environments, which can evoke emotional and physiological responses. Exposure to nature may trigger childhood memories and foster a sense of familiarity, calm, and safety [36]. The concept was further elaborated in collaboration with Stephen R. Kellert [37], who described how nature contact can meet a range of human needs, such as security, learning, meaning, and aesthetic experiences. The hypothesis distinguishes between direct contact (being physically present in nature), indirect contact (e.g., indoor plants or images of nature), and symbolic contact (e.g., metaphors or narratives), all of which can promote human well-being [37].

Kaplan and Kaplan’s Attention Restoration Theory (ART) [38] describes nature’s capacity to restore mental energy after periods of cognitive fatigue. According to the theory, human attention comprises two types: directed attention and involuntary (spontaneous) attention. Directed attention requires deliberate focus and the filtering of information, which is mentally demanding, potentially fatiguing and typically associated with complex and overstimulating environments such as cities, traffic, or office spaces. In contrast, involuntary attention is effortless and is especially activated in natural environments [38]. According to this theory, nature stimulates “soft fascination,” where attention is gently captured without conscious effort. This includes mild and undisturbed sensory impressions such as the sound of waves, wind rustling through trees, or birdsong. These sensory experiences activate involuntary and spontaneous attention, as they are perceived as pleasant and do not demand deliberate focus, cognitive control or decision-making. According to Kaplan and Kaplan, nature thus provides a restorative process for the brain by offering a break from directed attention and its associated demands. Nature invites reflection and inner calm, thereby serving as a source of mental recovery and renewed energy [38].

## 2. Materials and Methods

### 2.1. Design

We conducted qualitative anthropological fieldwork [39] in Denmark in April 2025 consisting of participant observation and semi-structured interviews with five people diagnosed with ADHD who engaged in winter bathing. Prior to these interviews, we carried out a pilot interview with another person diagnosed with ADHD who also practices winter bathing, and we also observed and participated actively [40] in winter bathing together with a group of people with ADHD and autism in a winter bathing club named HICE (Heat, Ice, Community & Exercise). In this club, the participants practiced cold-water exposure in a tub placed in nature, and all were professionally diagnosed with ADHD and/or autism. Finally, we conducted individual interviews with psychologist (K) and ADHD mentor (B), both of whom had professional experience working with individuals with ADHD who engage in winter bathing. The two professionals were included to gain a broader understanding, obtain more nuanced insights, and help address a knowledge gap identified during our research.

The reason for using a qualitative research design with individual interviews was to explore and understand the participants’ thoughts, feelings, and experiences related to winter bathing, thereby enabling an in-depth exploration and analysis of individual experiences. Our meeting with the psychologist took place in person, while the meeting with the ADHD mentor was conducted online. Prior to both meetings, we sent a set of questions, which served as the basis for the semi-structured interviews. The meetings lasted approximately 45–75 min.

At the winter bathing club HICE, we carried out unstructured observations [41], noting initial impressions and the general character of the setting, followed by structured observations [41] focusing on participants’ reactions and experiences in response to cold-water exposure. Finally, we conducted qualitative observation [41], with particular attention to the interaction between participants and their behavioural patterns. This approach aimed to gain a holistic understanding of the setting, the participants, and their interaction with each other during cold-water exposure. During our active participation in cold-water exposure, we also engaged in informal conversations with both participants and instructors.

To gain a detailed understanding of the participants’ behaviours, emotional states, and experiences, we conducted semi-structured interviews. The interview guide was developed based on a phenomenological approach supplemented by a hermeneutic approach [42,43,44]. It was centred around three main themes: ADHD, winter bathing, and subjective experience. It was designed in alignment with our research question. The interview guide for the participants is available in Appendix A and for the psychologist (K) and ADHD mentor (B) in Appendix B.

To include health professionals’ perspectives on the feasibility and potential implementation of winter bathing as an intervention for ADHD in the healthcare system, interviews were conducted with one ADHD mentor (B) and one psychologist experienced with ADHD (K). Both reported that they had clinical experience with winter bathing increasingly being used as an alternative strategy by individuals with ADHD to regulate the nervous system and promote a sense of bodily calm. The ADHD mentor (B), who has ADHD herself, described how winter bathing had helped her and several of her clients reduce racing thoughts, anxiety, and the associated sense of chaos. She experienced the cold water as a kind of “reset,” where the sensory system is momentarily overwhelmed, leading to increased mental clarity. She further described winter bathing as a tool for developing self-awareness and bodily presence. She described that many people with ADHD struggle to engage in seated mindfulness practices, and that the physical aspect of winter bathing makes it easier to become “present in the moment.” This was supported by clinical observations by the psychologist (K) of several clients beginning winter bathing and experiencing an improved mental state with a greater surplus of energy. According to K, winter bathing offers people with ADHD an opportunity to connect with their bodily boundaries. She explained that many individuals with ADHD struggle to sense when they are overburdened, and that winter bathing provides a clear somatic signal: “This is my limit.” Both emphasised that the effects may be individual and that winter bathing should be considered as a supplement to other strategies, not necessarily a replacement for medication. For some individuals, medication may have a limited effect, especially when stress levels are high, since elevated cortisol can inhibit the drug’s efficacy. In such cases, winter bathing may offer a meaningful strategy to activate the parasympathetic nervous system and lower stress. Finally, B pointed out that when one repeatedly confronts and overcomes discomfort (the ice-cold water), it can result in a sense of mastery and competence. This, in turn, may strengthen self-esteem, which is a challenge faced by many individuals with ADHD.

Prior to interviewing the participants, we conducted a pilot interview to practice and test our interview guide and role distribution (this was not included in the analysis).

In order to better understand and relate to the participants’ experiences, and upon consent, we actively participated [40] in winter bathing with four of the participants before conducting the interviews. This allowed us to take on the role of active observers, where participating in the winter bathing enabled us to gain a deeper understanding of the participants’ experiences. At the same time, we engaged as fellow human beings in their winter bathing routines, which strengthened our insight into participants’ perspectives and lived experiences [45]. Such engagement may enhance receptivity and empathy toward the participants’ shared experiences [46]. Through physical presence and the shared experience, a “tuning in” occurs for the observer’s mind–body, allowing observers to better pick up on the participants’ embodied responses, facilitating a deeper understanding of their experiences.

We prioritized conducting all interviews face-to-face. This enabled us to observe and engage in non-verbal communication before, during, and after the interviews, such as body language, facial expressions, and eye contact, which contributed to a sense of embodied presence in the conversation.

To create the best possible setting for the interviews, four were conducted in familiar surroundings at the participants’ usual winter bathing locations, and one took place in a meeting room at VIA University College in Aarhus N. We chose physical environments that were secluded and had good acoustics to optimize both focus and audio quality during recordings. These settings helped ensure calm and concentration for the participants, while also safeguarding their confidentiality.

Our study was primarily based on a phenomenological approach, supplemented by a hermeneutic approach [43,44]. We were curious about the phenomenon of winter bathing for people living with ADHD and aimed to explore this through personal experiences among five individuals with ADHD. Accordingly, our interviews were conducted with an open attitude, seeking to prevent our preunderstanding from steering the conversation [47]. The phenomenological approach supported our effort to listen attentively and to understand the experiences as they were lived by each individual.

We wanted the themes to emerge naturally from the empirical data and therefore chose to incorporate theory only after the interviews were completed. In this way, the participants’ experiences served as the foundation for our subsequent analysis.

During the analysis, we adopted a hermeneutic approach, using our understanding and knowledge to interpret the empirical data [43,44]. Along the way, we became increasingly aware of our own preunderstanding, which we actively used to create meaning and coherence. As our horizon of understanding expanded, we developed our hermeneutic spiral, deepening and refining the understanding with each pass. Our initial understanding was continuously adjusted in light of new insights from the data, and this revised understanding in turn influenced further interpretation. This approach was central to our attempt to both listen openly and to understand the participants.

### 2.2. Preunderstanding

In relation to our primarily phenomenological approach, and as emphasized by Malterud [47], we explicated our preunderstanding to set it aside in order to approach the empirical data and participants with openness and curiosity. As physiotherapists with a background in health sciences, our preunderstanding was shaped by both professional knowledge and general societal awareness of the topics of ADHD and winter bathing. We were aware that ADHD is increasingly being diagnosed and is often associated with symptoms such as impulsivity, hyperactivity, and difficulties with concentration.

We understood ADHD as a neurodevelopmental condition and recognized that individuals with ADHD may face challenges in relation to societal norms and expectations. Based on our knowledge, we understood that people with ADHD are generally at higher risk of achieving lower levels of education and income and being in conflict with the law. Furthermore, it was our preunderstanding that medication is often the primary treatment strategy for ADHD. Regarding winter bathing, the authors had varying levels of personal experience, ranging from beginners to several years of practice. We perceived winter bathing as potentially having a positive impact on mental well-being, including stress reduction. We understood it to involve a range of physiological responses, such as increased release of endorphins and dopamine, which may contribute to feelings of joy, relaxation, and pain relief.

We perceived winter bathing as a form of mindfulness with focus on breathing and presence. Although the cold water may be perceived as uncomfortable by some, the activity is easily accessible in Denmark and has grown in popularity in recent years. We held the impression that many winter bathers are resourceful individuals, and we were aware that winter bathing is a seasonal activity not available year-round. Furthermore, we were aware of professional disagreements and uncertainty regarding the physiological effects of winter bathing, as research in this area is ongoing and no clear scientific consensus has yet been established.

### 2.3. Entering the Field

To recruit relevant participants for the project, we compiled a list of the 20 largest winter bathing clubs in Denmark [48]. We then contacted these clubs via email, presenting our project and requesting permission to share a recruitment post in their Facebook groups (Appendix C). The aim was to reach adults with ADHD who might be interested in participating in an interview about their experiences with winter bathing.

Initially, we defined the following inclusion criteria for participation: diagnosed with ADHD; age 18 or older; and regular engagement in winter bathing.

The recruitment post resulted in 19 responses via email, Facebook, and SMS. We declined further inquiries after reaching more than the desired number of participants. Consequently, we decided to refine the selection criteria, as it was not possible to include all interested individuals. The additional criteria were a minimum of four months’ experience with winter bathing; winter bathing at least once a week; no prior relationship with the research group; and engagement in winter bathing in natural settings such as the sea, lakes, or fjords.

These criteria were applied to ensure that participants had sufficient experience with winter bathing and could contribute relevant and in-depth perspectives to our research question. Due to limited time, we selected five participants. When selecting participants, we aimed for a heterogeneous group in terms of gender, age, location, and winter bathing routines. This included variation in whether participants were winter bathing through winter bathing clubs or independently, as well as whether they bathed in the sea, lakes, or fjords. Additionally, we sought to apply a first-come, first-served principle in the selection process.

### 2.4. Ethics

This study was conducted in accordance with the Declaration of Helsinki [49]. The design, aim, and procedures were evaluated and approved by the Central Denmark Region Committee on Health Research Ethics, Skottenborg 26, DK-8800 Viborg, Denmark (record number: 1-10-72-9-25).

In accordance with the Declaration of Helsinki [49], we obtained voluntary informed consent from all participants. Prior to each interview, participants signed a consent form, which provided relevant information about the purpose of the project and data usage, and clarified that participation was voluntary and that consent could be withdrawn at any time without consequences [50]. At the beginning of each interview, the purpose and our identities and roles were clearly communicated.

During the transcription process, participants were anonymized and assigned the letters A, B, C, D, and E to protect their identities and ensure confidentiality [41]. In addition, we used people-first language to treat participants as respectfully as possible by emphasizing the person rather than the diagnosis [51].

None of us had any prior relationship with the participants. For ethical reasons and in the interest of transparency, we disclosed our background within physiotherapy to help build trust and ensure participants felt comfortable with who they were sharing their information with and how it would be used. We acknowledge that this background may have influenced how participants responded during the interviews.

### 2.5. Data Collection

All semi-structured interviews were recorded using two audio recorders, both secured with password protection to ensure participant confidentiality. The recordings were transcribed using the transcription tool in Microsoft Word, which is protected by two-step verification. To ensure consistency and uniformity, we used transcription rules such as writing “X” for specific names and places and “...” for longer pauses, and we reviewed all transcripts by listening to the recordings and adjusting the transcriptions to reflect the spoken language as accurately as possible. The software automatically omitted filler words such as “uh” and “mmh.” In cases of rapid speech, the playback speed was adjusted to ensure precision in transcription.

Each interview was conducted by two of us as interviewers to facilitate a conversational flow and ensure that all questions were addressed. One person participated as an observer and had the opportunity to ask follow-up questions at the end. We rotated roles for each interview, allowing all researchers/authors to gain experience as both interviewer and observer.

### 2.6. Thematic Analysis of the Empirical Data

Our perspective was phenomenological, which involved temporarily setting aside preunderstanding notions, and the empirical data from five interviews were thematically analysed using Malterud’s four-step approach, based on Giorgi’s psychological phenomenological thematic analysis [47,52]. The process followed four phases: (1) total impression—from chaos to themes; (2) identifying and sorting meaning units—from themes to codes; (3) condensation—from code to meaning; and (4) synthesizing—from condensation to descriptions and concepts to provide a structured and transparent approach to achieving a deeper understanding and interpretation of the data.

The analysis was conducted inductively to ensure an open, data-driven approach in which theoretical concepts were not applied in advance to guide interpretation. This allowed themes to emerge directly from the empirical data, free from preconceptions [47,53,54]. The transcribed interviews were analysed across participants to identify both similarities and differences in their statements. The data were read multiple times to develop a thorough understanding and to avoid allowing our preunderstandings to influence the coding and thematization process.

In the first step, the transcripts were read through to form an overall impression. Following the individual readings, we applied researcher triangulation [55], where we collaboratively discussed and compared our preliminary themes. Preliminary Themes: Happy; Well-being; Always a Success; Mental calmness: peace of mind and relief from racing thoughts; Winter Bathing as a Supplement to Medication; Anxiety and Depression; Works Every Time; Nature; The Body; Coping Strategy.In the second step, the interview data were reviewed line by line by the entire research group, with a focus on identifying meaning units. These units were color-coded and linked to the themes developed during step 1.

During this process, it became apparent that some of the initial themes overlapped or were too narrow in relation to our research question. As a result, the theme “*Well-being*” was merged under the broader theme “*Happy*”, and the theme “*Works Every Time*” was excluded due to insufficient content to stand alone.

This comprehensive review resulted in seven coding groups, which were used in the subsequent stages of analysis. Coding Groups: Joy; Mental calmness: peace of mind and relief from racing thoughts; Winter Bathing as a Supplement to Medication; Anxiety and Depression; The body; Coping strategy; Nature. At step three, we reviewed the meaning units and sorted them into subgroups within each coding group. We then condensed the content so that the essential points were clearly presented, either as brief, summarized text segments or as merged statements conveying the same meaning. A few powerful quotes were selected and retained in their original form as golden quotations [47,52]. During this process, we excluded data that were not relevant to our research question, and some units were reassigned to more appropriate coding groups. We also renamed the coding groups to better reflect the participants’ experiences and the context of the project. For example, “Joy” was renamed “Joy: sustained positivity”; “Winter bathing as a supplement to medication” and “Anxiety and depression” were combined under “Winter bathing as a supplement or alternative to medication for anxiety, depression, and ADHD”; “The Body” became “Bodily awareness and connection to the body”; “Coping strategy” became “Coping with and managing everyday life better”; and “Nature” was renamed “Nature: essential for motivation and calm.”

At step four, we re-contextualized each theme for an academic text. The condensed meaning units were compiled and written into an analytical narrative for each coding group, presenting the essence of the participants’ statements in a clear and structured manner. We reformulated the text from first person to third person to establish analytical distance and ensure a more neutral presentation. Along the way, we integrated selected golden quotations that best supported the coding groups. Some quotes were later replaced if more precise or relevant ones were identified.

The outcome of this phase is the thematic findings presented in the Results section. The final themes, which also serve as subheadings in the Results section, were: (1) Mental calmness: peace of mind and relief from racing thoughts; (2) Bodily awareness and connection to the body; (3) Joy: sustained positivity; (4) Coping with and managing everyday life better; (5) Winter bathing as a supplement or alternative to medication for anxiety, depression, and ADHD; and (6) Nature: essential for motivation and mind–body calmness. Subsequently, we analysed the empirical data using relevant theoretical perspectives.

### 2.7. Analytical Perspectives

We were very conscious about the balance between the empirical data and theory, of not allowing the theoretical approach to overrule the empirical data, and above all, of allowing the empirical data to step forward without too much theory, as, for example, underlined by Wolcott [54] and Bundgaard [53].

Therefore, at first, we analysed the empirical data, and *after* this, we discussed our results with the theoretical perspectives of Merleau-Ponty [28,29], Antonovsky [30], Bandura [31], Deci and Ryan [32,33,34,35], Wilson and Kellert [36,37] and Kaplan and Kaplan [38], as introduced in the introduction section.

## 3. Results and Analysis

### 3.1. Participants

The participants consisted of five individuals—two men and three women—diagnosed with ADHD, aged between mid-30s and late 50s, who engaged in winter bathing. There was variation in both geographic location and winter bathing practices: two participants bathed in the sea, two in fjords, and one in a lake. Four of the participants were members of a winter bathing club, while one bathed independently. Regarding medical treatment for ADHD, there was also variation: three participants used medication regularly, one used it as needed, and one did not use medication at all. The interviews lasted between 34 and 75 min, with three interviews lasting approximately 60 min. Because the participants were able to dress and winter bathe, they were much less likely to have acute, severe depression. To ensure anonymity, the participants were presented as a collective group while highlighting key variations within the group. We did not know the participants prior to the study.

### 3.2. Themes

Six main themes were identified from the data: (1) Mental calmness: peace of mind and relief from racing thoughts; (2) Bodily awareness and connection to the body; (3) Joy: sustained positivity; (4) Coping with and managing everyday life better; (5) Winter bathing as a supplement or alternative to medication for anxiety, depression, and ADHD; and (6) Nature: essential for motivation and mind–body calmness.

#### 3.2.1. Theme 1: Mental Calmness: Peace of Mind and Relief from Racing Thoughts

A shared theme among all participants was the experience of mental calmness with peace of mind and relief from racing thoughts after winter bathing. They described it as a mental reset, where intrusive thoughts faded, their minds felt lighter, and they became more present in the moment:

*B: “Having ADHD often means having lots of thoughts that just take over, but when I go into the water, it’s like they disappear somehow, because you can’t think of anything else... it resets me.”*

*C: “It narrows down the 44 highways I have in my head.”*

Four participants highlighted that this sense of calm improved their concentration and ability to focus on one task at a time:

*A: “I can handle things better. I’m more focused on what I’m doing.”*

*A: “It’s more in my head. I can focus on details I never noticed before.”*

They explained that everyday tasks like cooking, reading, or completing projects, previously overwhelming, became more manageable. Three participants noted the immediate calming and stress-reduction of winter bathing and intentionally used it as a strategy:

*E: “If only I had gone for a dip, it would’ve been much easier.”*

*D: “I can really feel it right away when I get out.”*

We observed that one participant spoke noticeably more slowly after winter bathing compared to before. The tempo was calmer and more balanced, which could be interpreted as a sign of the inner calm the participant described.

Three participants reported that winter bathing brought them a sense of well-being and calm they could not achieve through other methods. This tranquillity also had a positive effect on their sleep. Two stated that they fell asleep more easily and had better sleep quality:

*C: “Yes, then there’s calm. I relax. I fall asleep quickly at home.”*

*A: “Sometimes I even take a nap during the day, that’s how relaxed I feel afterward... I might lie down and read or something.”*

Overall, participants described winter bathing as an effective activity for calming the mind, increasing focus, enhancing mental well-being, and improving sleep.

According to the perspectives of Merleau-Ponty [28,29], the body and mind are inseparably connected, with the body being an anchor to the present moment. The body is the closest one can come to the now [28]; the body is the present’s place, and the body can (re)establish contact with the mind, bringing the mind back to the present [56]. The body acts and senses pre-reflectively, as seen in participants’ experiences of cold-water exposure through winter bathing and immersion in nature. The participants described how winter bathing created a deep bodily awareness and connection to the body that grounded them in the present moment and created peace of mind and relief from racing thoughts by quieting racing thoughts and resetting the mind. We assessed that this state was connected to reflective awareness, where increased concentration and focus made it easier to direct attention toward everyday activities, such as cooking, reading, and completing projects. More specifically, this was observed in one participant, who, after winter bathing, spoke more calmly and in a more balanced manner, as an expression of inner calm that arises when the body acts and senses pre-reflectively, and the mind is then given space for reflection.

The participants stated that they experienced a sense of mental well-being and calm that they were unable to find through other methods, which, according to Antonovsky’s theory of Sense of Coherence (SOC) [30], can refer to a sense of manageability in coping with everyday life. The experience of winter bathing appeared to the participants as repetitive and recognizable, which created a sense of comprehensibility. They described an immediate effect of reduced stress and perceived winter bathing as a valuable tool for creating peace of mind and relief from racing thoughts. It provided increased focus, improved sleep, and mental well-being in daily life. Although it could be challenging, winter bathing was experienced as meaningful and worth engaging in.

#### 3.2.2. Theme 2: Bodily Awareness and Connection to the Body

Four participants described how winter bathing heightened their bodily awareness and provided a sensory experience that fostered a connection to the body:

*B: “You have to be very focused on your body when entering the cold water. First of all, you need to think about your breathing and stay connected to it. When stress, depression, and anxiety are often associated with ADHD, the water is just really good, because you’re forced to be completely present in your entire body.”*

The physical sensation of being surrounded by water and the piercing cold against the skin made them acutely aware of their bodies and prevented other thoughts, providing a feeling of being in the present moment. Remaining in the cold water required increased attention to breathing. One participant explained:

*B: “When I’m in the water and feel the cold and the stinging on my skin, I have to be present in my body, relax, and control my breathing, to feel my body and myself.”*

At the same time, four participants described how the sensation of a relaxed body led to a deep mental calm and a sense of well-being. Bodily elements such as breathing and physical relaxation were highlighted as central factors contributing to mental well-being:

*C: “This, this is well-being. It’s balm for body and soul … I have no doubt about that.”*

Several participants described that after being in the cold water, their ability to feel their body positively affected their mental well-being. One participant expressed:

*E: “For some, it’s clearly this interaction, where you can feel the body regulating itself. I definitely think that’s the therapeutic part – that you sense your body is regulating, you learn to feel your body, and it does something for your psyche.”*

For participant B, this bodily awareness and connection to the body served as a counterbalance to a life marked by racing thoughts and depression:

*B: “I feel my body much better now, and I think that’s really good for me, someone who spends so much time in my head, that there must be some kind of balance. I also think that the more I can be in and feel my body, the less I need to be in my head.”*

This experience was also observed during fieldwork, as one of the participants appeared physically calmer and more relaxed after winter bathing. This observation supported the participants’ statements about bodily calm and mental well-being immediately following winter bathing. For the participants, winter bathing was not merely a break from everyday life but a bodily presence that supported mental well-being and transformation. The connection between body and mind appeared to be essential to the experience.

In line with Merleau-Ponty’s understanding of the inseparable connection between body and mind [28,29], the participants described how winter bathing strengthened this connection. They felt their bodies in the cold water, while racing thoughts subsided and calmness settled in. One participant described this clearly as a physical regulation with a positive impact on the psyche, while another explained how bodily sensations helped them get out of their head and find peace. According to Merleau-Ponty, being present in one’s body enhances connection to the present moment, fostering presence [28]. This was reflected in the narrative of a third participant who described winter bathing as well-being and balm for body and soul. Based on the theory, winter bathing appears to facilitate bodily awareness and presence in the moment, leading to peace of mind and relief from racing thoughts and presence, and enhanced well-being. The pre-reflective body senses the stinging cold on the skin and responds through breathing, which heightens bodily awareness and connection to the body, brings about deep mental calmness, and a sense of well-being. In this way, the participants experienced mental well-being through bodily sensations in the cold water.

#### 3.2.3. Theme 3: Joy: Sustained Positivity

All participants mentioned that winter bathing made them feel happy. The joy they experienced was described using expressions such as being completely joyful, a rush of happiness, and euphoria:

*E: “It gives a kind of euphoria. I feel uplifted and get a sort of kick from it, and it lasts, and then I ride on that feeling for a long time.”*

The joy was not merely momentary, as it remained with the participants afterwards. They reported feeling happier when returning home from winter bathing, which elevated their mood. For some, it lasted the rest of the day, while for others, it continued for several days. Four participants also mentioned that they had become more positive after they had been winter bathing. They described this as increased optimism, feeling more positive, and less melancholic:

*D: “I get so happy. I can’t state this enough, I become so cheerfully happy. It’s crazy. It’s the best thing that has ever happened to me, apart from when I had my youngest son.”*

Three participants expressed that the joy and positivity occurred every single time they went winter bathing. They described it as consistently being a positive experience:

*A: “It’s always a success when I’ve been out here.”*

The joy and positivity were not only experienced by the participants themselves. Two participants shared that their family members had also noticed a change. Winter bathing thus appeared to have a stable and lasting positive effect on the participants’ mood and mental well-being. According to both the participants and their relatives, the activity contributed to increased zest for life and a more positive attitude in everyday life.

The participants’ descriptions of joy and increased zest for life can be understood through the Self-Determination Theory (SDT) [35]. As the participants chose to engage in winter bathing voluntarily, their sense of autonomy and self-determination were strengthened. Several participants mentioned the joy of bathing within the community of a club, and family members noticed a change in their mood following winter bathing, which supported a sense of belonging both within and beyond the club environment. This aligns with the theoretical assumption that fulfilling these psychological needs can foster greater intrinsic motivation and thriving, which may help explain the sustained joy and positive influence on the participants’ mood and mental health.

The participants’ experiences of joy and sustained positivity can also be interpreted through the biophilia hypothesis [37], which posits that humans possess an innate drive and need to seek connection with nature. When the participants engaged in winter bathing, they were in direct contact with natural elements such as air, wind, and water, which enhanced their sense of well-being. One participant even compared the joy of winter bathing to the experience of having a child. Another described winter bathing in nature as a guaranteed success, emphasizing that it was always a positive experience. The physical and mental connection with nature appeared to strengthen their mood, and one participant stated that this feeling lasted for several days. Several participants also noted that their families observed this change, which reinforces the idea that nature has a long-lasting impact on their mental well-being.

#### 3.2.4. Theme 4: Coping with and Managing Everyday Life Better

Four participants stated that winter bathing provided them with surplus in their daily life, emotional stability, and increased focus, all functioning together as a strategy that made it easier for them to carry out their daily activities and made the day feel more manageable:

*D: “Then I can really handle everything better.”*

*B: “There are some things that become easier to master, and then I know that the water is always there, so I can always go out into it again if I need more.”*

*E: “It has, in a sensory way, given a small embrace to the rest of the day. So now the body has had a shock, and then the world might be a little gentler to approach.”*

One participant explained that the feeling and control derived from the calming experience of winter bathing were not limited to the specific moment but could be transferred to other situations, helping to create the necessary calm to overcome them:

*B: “The feeling I get from the cold water I can almost bring it into other situations. It’s as if it can be activated elsewhere if I need it, because I know how it feels to enter cold water. It calms me.”*

Overall, the participants’ statements indicated that winter bathing not only created immediate well-being but also functioned as a tool for emotional regulation and mental coping. It became easier to manage and sustain everyday life as well as to control emotional reactions. This experience of coping was described as dependent on regular bathing, as several mentioned that they only noticed the difference when bathing two to three times per week. As explained, the benefits were not limited to the specific moment in the cold water; they were transferred to many other situations in everyday life, and, in order to maintain the benefits of winter bathing, the participants engaged in cold-water bathing on a weekly basis, i.e., at least once per week.

The participants’ accounts of being able to manage and handle everyday life better correspond to Antonovsky’s [30] description of coping, which promotes health. This was expressed through the participants experiencing mental calm, more surplus in everyday life, and increased focus, providing them with a sense of structure and comprehensibility. Manageability was reflected in the participants’ perception that winter bathing granted them access to resources such as joy and emotional stability, which helped them maintain a balance between demands and resources. Meaningfulness was present as winter bathing was experienced as valuable even when it could be challenging. One participant explained how the feeling from winter bathing could be recalled and “activated” in other situations, serving as a coping strategy to find calmness and clarity in everyday life.

According to Bandura’s theory of reciprocal determinism [31] (personal factors, behaviour, and environment), mental well-being, emotional control, and the ability to handle everyday life are interconnected and mutually influence one another. Personal factors such as peace of mind and relief from racing thoughts, surplus in everyday life, increased focus, and joy influenced the participants’ behaviour in the form of improved emotional regulation and control. This enabled the participants to engage with their environment, such as family and friends, and to manage everyday life better with a sense of mastery.

#### 3.2.5. Theme 5: Winter Bathing as a Supplement or Alternative to Medication for Anxiety, Depression and ADHD

Four of the participants reported that winter bathing had reduced their symptoms of anxiety, depression, and ADHD, respectively, and that they used winter bathing as a supplement or substitute for their medication:

*B: “I already knew that the water would relieve some of the anxiety.”*

*B: “While I have been winter bathing, in fact, I have not had any depressions.”*

*D: “It actually does the same as ADHD medication does, just without the side effects.”*

Two of them stated that winter bathing had helped them through difficult periods characterized by a lack of desire to live. One described it as follows:

*B: "For me it has had a healing effect on my depression. I have been all the way down, to the point where I did not want to live anymore, two years ago.”*

In addition to alleviating depressive thoughts, two participants stated that they use winter bathing as a replacement for medication due to the side effects they experienced from the medication, while two others use winter bathing as a supplement to their medication. One of the participants said:

*D: “I truly believe that instead of prescribing antidepressants (medicine), they should be sent into the cold water, because that can relieve it.”*

Furthermore, three participants reported that winter bathing had reduced their need for ADHD medication, with one participant explaining that winter bathing is a supplement and a strategy that helps in managing everyday life with ADHD:

*D: “It has more than halved my need for medication, as it brings calm to my mind and soul.”*

One participant described how winter bathing helped during difficult times and replaced the use of self-medication with drugs. Another stated that medication is important in managing ADHD and that the combination of medication and winter bathing is a positive strategy, as both help but work in different ways:

*B: “Medication is not a solution to everything. Medication is the scaffolding for ADHD.”*

Overall, the participants experienced that winter bathing reduced their symptoms of anxiety, depression, and ADHD, and two participants used it as a substitute for medication, while two other participants used it as a supplement to their medication. All used winter bathing as a way to better manage and cope with everyday life.

According to Antonovsky’s theory of SOC [30], comprehensibility and manageability were expressed among the participants, as winter bathing strengthened their sense of being able to master everyday life. Several stated that winter bathing reduced symptoms of anxiety, depression, and ADHD to such an extent that some of them opted out of medication, while others used winter bathing as a supplement. Winter bathing thereby became a concrete and comprehensible strategy to create balance in everyday life, generating significant value and meaningfulness for the participants.

Winter bathing as a supplement or alternative to medication for managing severe mental conditions was something the participants chose themselves and experienced the effects of, which created a sense of control and autonomy. The competences arose through experiences of the positive effects of winter bathing, reducing their symptoms. The winter bathing club provided the framework for creating a pleasant place where the participants felt a sense of belonging. According to SDT and the three psychological needs [35], winter bathing promoted intrinsic motivation as a strategy to handle challenging mental conditions, either as a supplement or an alternative to medication. When winter bathing was experienced as a self-chosen alternative or supplement to medication, it fostered a sense of mastery and contributed to improved thriving, potentially with a reduced need for pharmacological treatment.

#### 3.2.6. Theme 6: Nature: Essential for Motivation and General Mind–Body Calmness

A common theme among all participants was that elements of nature, such as the sea, fjord, forest, sky, sun, moon, fresh air, and open spaces, played a central role and constituted an essential aspect of the winter bathing experience. The participants emphasized that cold-water exposure, for example in a domestic setting such as a tub, could not be compared and would be demotivating, as part of the experience was in the aesthetics and the sensory context that nature provided. One participant described it as follows:

*E: “It has been most special when it is really cold, and the view is incredibly beautiful by the sea, completely still, and the sun has just begun to rise, full moon, or when there is a certain special atmosphere in nature.”*

Furthermore, two participants stated that the view with an open sky was significant. One described:

*C: “The water is important, and the open sky and the clouds”*

Nature provided two participants with a soothing sense of general calm and well-being. One described it as a truly wonderful experience to jump into the water by the forest, and compared the calm from winter bathing in nature metaphorically to the calmness of an open fire:

*C: “Nature gives you peace, just like fire gives peace. I think it is something we all know. It simply gives peace, just like sitting and watching a crackling fire.”*

Another described it as a great sense of well-being to be surrounded by nature, gazing at the horizon, immersed in the cold water, and being in the present moment, which paused the racing thoughts. Being in nature and surrounded by the sea evoked a fundamental sense of general calm, which the participant described as a so-called primal calm. The combination of being surrounded by nature and in cold water was described by one participant as elements that contributed to a physically relaxed state that lasted for the rest of the day:

*E: “When I am sitting on a bathing jetty and looking at the water, it is very much the element of water that does something for me. But when I am in it, I become completely refreshed. I went winter bathing this morning, and I can feel that my body is relaxed for the rest of the day.”*

Overall, the participants experienced nature as an essential and motivating part of winter bathing, which provided them with calmness and well-being. One participant described it as simply as:

*A: “Nature is my medicine.”*

Winter bathing can be understood through SDT [35], as the participants described it as a voluntary and self-chosen activity, which points to a strong sense of autonomy. The physical experience of enduring the cold contributed to a sense of competence, while the encounter with nature strengthened a sense of relatedness. When these three psychological needs were fulfilled, intrinsic motivation was promoted. This may help explain why winter bathing in nature felt motivating and provided lasting joy.

The participants’ positive descriptions of nature can be understood through the concept of biophilia [37], the idea that humans have an innate tendency to seek closeness and contact with nature. When the participants referred to the sea, the sky, and the light as “medicine” or spoke of a primal calm, it indicated a deep-rooted connection to the natural elements that provide calmness and security. According to this hypothesis, contact with nature has a positive effect on both emotional and physiological responses, which aligns with the participants’ perception that nature was not merely a setting for winter bathing but an important factor for joy, familiarity, safety, and aesthetic experiences.

The calmness, well-being, and closeness that several participants described experiencing in nature can be understood through ART [38]. The participants’ narratives about natural surroundings such as the sea, forest, and horizon fall under what the theory calls “soft fascination,” where attention is captured in a gentle way without requiring mental effort, inviting spontaneous awareness. The participants experienced the encounter with nature as both strengthening and restorative, with one describing nature as medicine.

## 4. Discussion

The aim of this study was to explore how winter bathing was experienced to affect the mental well-being of five adults diagnosed with ADHD. Our main findings were that winter bathing improved the participants’ mental well-being, with an immediate and long-lasting general mind–body calmness and relief from racing thoughts, peace of mind and increased concentration and focus. Winter bathing enhanced bodily awareness and mind–body balance/connectivity with a felt connection with the body and the present moment. Winter bathing promoted persistent positive emotions with profound feelings of joy and happiness, and a prolonged sense of uplift. These emotions were associated with improved mood, energy, emotional regulation and overall zest for life. Winter bathing strengthened the ability to cope with and handle everyday challenges, and most of the participants experienced a transfer of the general mind–body calmness, benefiting them across situations in everyday life. Nature and nature connectedness appeared to be an essential element for motivation and general mind–body calmness. Finally, winter bathing functioned as a supplement to, and for some participants as an alternative to, medication for anxiety, depression, and ADHD. Participants reported a reduced need for medication and some no longer required medication at all.

While ADHD is clinically characterized by difficulties in maintaining routines and executive functioning, and given that winter bathing requires significant planning, motivation, and tolerance of discomfort, it has to be emphasized that the participants explained that they managed to maintain this demanding routine simply because they were strongly motivated both by the joy of the winter bathing in itself and by all the benefits achieved through the winter bathing.

Our findings are important, as they provide deeper insight into the lived experiences of individuals with ADHD who engage in winter bathing. Furthermore, since evidence for non-pharmacological interventions in adults with ADHD remains limited [4,5,10,11], these findings support the potential of winter bathing as a non-pharmacological intervention for this group.

Supporting our results, the HICE participants described that doing cold-water exposure in tubs surrounded by nature provided them with a break from racing thoughts, improved mood, a sense of bodily awareness and connection with the body, general mind–body calmness, and reduced stress with surplus in daily life and structure for the rest of the day. These findings were also emphasized by one of the instructors from HICE, who specifically described that she/he observed similar changes in the participants after a 12-week program. Furthermore, we also observed that several of the HICE participants displayed more relaxed body posture, smiled more, listened more calmly, and were more focused compared to before the cold-water exposure. These additional observations strengthen the confirmability of our findings.

Moreover, our findings were supported by descriptions from psychologist K and ADHD mentor B. For instance, B described how the cold water “resets” and provides mental clarity, and how winter bathing functions as a tool to strengthen self-awareness and bodily presence. We consider this perspective essential, as it points to winter bathing as a practice that can support mental well-being for people with ADHD. This is clearly reflected in our themes “*Mental calmness: peace of mind and relief from racing thoughts*” and “*Coping with and managing everyday life better*”. Both K and B also described that for their clients with ADHD, winter bathing can promote increased focus and well-being, which supports one of the themes in this study, “*Joy: sustained positivity.*” Finally, K emphasized that winter bathing helps people with ADHD to sense their bodily boundaries, which corresponds with our theme, “*Bodily awareness and connection to the body.*”

It is important to underline that both K and B pointed out that winter bathing should be considered a supplement to other strategies and not necessarily a replacement for medical treatment. We find it noteworthy that several of our participants reported experiencing “*Winter bathing as a supplement or alternative to medication for anxiety, depression, and ADHD,*” which, together with the experiences of the two professionals, indicates that further research on this topic will be valuable. Overall, source triangulation between our findings and the descriptions from K, B and the HICE participants strengthens the confirmability of our results, suggesting relevant mechanisms for how winter bathing may enhance mental well-being for people with ADHD.

Our findings of experiencing winter bathing as a meaningful activity that supported mental well-being for five adults with ADHD raise the question of whether other non-pharmacological interventions might also have similar effects. An example of this is mindfulness, which was examined in a pilot study by Mitchell et al. [57]. The study showed that adults with ADHD, after participating in an eight-week group-based mindfulness program, experienced a significant reduction in ADHD symptoms, improved executive functioning, and better emotional regulation. This suggests that activities such as mindfulness may offer some of the same benefits as winter bathing for individuals with ADHD, with respect to emotion regulation, reducing racing thoughts, and improving the ability to manage everyday life. A systematic review from 2023 [58] concluded that novel treatment methods such as transcranial direct current stimulation (tDCS) and the combination of medication and cognitive behavioural therapy both appeared promising, although further well-documented studies are needed.

The Danish Health Authority has published a new national clinical guideline for the treatment of ADHD [5]. This guideline recommends non-pharmacological intervention in addition to medication or as an alternative to medication for individuals not using medication. Examples of non-pharmacological interventions are cognitive behavioral therapy; dialectical behavior therapy; a combination of the two; mindfulness; mindfulness-based cognitive therapy; and cognitive training or psychosocial interventions [5].

The present study demonstrated that winter bathing, as an embodied experience in nature, had a positive impact on the participants’ mental well-being and contributed to their ability to better manage everyday life. This can be understood from a biopsychosocial perspective, where biological, psychological and social factors are regarded as closely interconnected [59]. The impact of nature connectedness on mood, motivation and general mind–body calmness was also emphasized by two qualitative studies. In a nature-based mindfulness study in Denmark [60], nature connectedness experiences were associated with gratitude, a sense of coming home, and the sense of connection with oneself, others and nature, as well as the sense of being away from everyday life and immersed in nature, were the most emphasized qualities supporting the capacity for self-regulation. In a blue space follow-up study with freedom on water through Stand-Up paddling [61], it was evident that physical activity outdoors in blue nature reduced negative emotions and improved mental health and overall well-being, with feelings of calmness and a sense that everything will be all right, positivity, grounding, healing, free space, and freedom. As one of the participants from the Stand-Up paddling study [61] said, “Those days where I’m really struggling with my disease, I go out and feel the nature as a calm and peaceful place, and I just listen to the birds”.

Our findings are supported by a previous study on winter bathing published in 2024 [16]. Together, the 2024 study and the present study reported how winter bathing provided a sense of calm and helped reduce racing thoughts. Furthermore, both studies found that winter bathing helped the participants cope better with everyday challenges. Several participants described how the calmness and lasting joy they experienced from winter bathing helped them better manage everyday life. For example, one participant mentioned being able to recall the feeling from the cold water in other situations as a way to handle daily challenges. The significance of this is apparent, and thus winter bathing may hold a strong potential to promote quality of life, including for individuals living with ADHD.

The 2024 study also described social interaction as an essential part of the experience with winter bathing, which our study did not highlight. One possible explanation could be that our participants were already challenged by racing thoughts and therefore had a greater need for calmness and presence than for the social aspect of winter bathing. This variation is interesting, suggesting that different mediating mechanisms are involved for different groups and thus can potentially have varying importance when considering winter bathing in different clinical and non-clinical groups. Additionally, we found that some participants used winter bathing as a way to manage symptoms of anxiety, depression, and ADHD, either as a supplement or an alternative to medication. This was not emphasized in the 2024 study and may represent a specific and important perspective for people with ADHD that points to winter bathing as a complementary way to regulate emotions and find balance in everyday life. Finally, both studies showed that nature played an important role. Both studies described how the natural surroundings contributed to creating calm and well-being. It was not only the cold water that made a difference, but also the whole experience of being in nature, which offered a respite from the busyness of everyday life.

Merleau-Ponty’s perspectives on body and mind inseparability and the body being an anchor to the present moment underline our results concerning how winter bathing through bodily sensations and bodily awareness created presence in the moment and a break from racing thoughts for the participants. According to Ponty [62], the body is regarded as an *anchor to the now,* where individuals are at one with their body and thereby fully present. The body then makes it possible to be fully present in the moment, free of thinking about the past and the future [62]. Our body is the closest we can come to the present [28], and thus our body is the *place for the present*. From its position in the present, the body can (re)establish contact with the mind, bringing the mind back to the present [56]. Theories by Merleau-Ponty underline the importance of including the body in the treatment of mental challenges. Even with the profound understanding of body and mind being inseparable and affecting each other reciprocally [62,63], we do not have a single word in the West that encompasses both mind and body. This creates a linguistic challenge in avoiding dualism, due to the need to specify both. The very act of articulating mind and body separately, however, presents a dilemma, because the wording itself can signal that the body is something other than the psyche or the mind, which unfortunately signals dualism. We all (including the participants) are products of our time and culture and are therefore also influenced by the dominant role of biomedicine in the healthcare system (in Denmark, in the Nordic countries, and in the West) [64]. In this study, regardless of language use, our understanding is that the mind and body are inseparable and are both intra-connected and intra-influencing themselves as a whole mind–body entity.

Health professionals and health workers with appropriate skills are central to the expansion of mental health services and to better outcomes [65]. By integrating mental health into general health, disease-specific and social care services, an important opportunity to manage mental health problems better, promote mental health and prevent mental disorders is provided. As agreed upon in the Comprehensive Mental Health action plan by the WHO, all 194 member states have committed themselves to actions that promote mental health. This includes task-sharing, whereby general healthcare professionals and actors from civil society become more involved in efforts to promote mental health. This includes equipping health workers with training in mental health and interventions to promote mental health and manage mental disorders among the individuals they see, in addition to providing general wellness information and screening for health-related conditions [65]. It will probably imply a re-definition of health workers’ roles, as agreed upon among the member states, and result in changes to the existing service culture and attitudes of general health workers, social workers, occupational therapists, physiotherapists and other professional groups. It will also require the achievement of new knowledge and skills to identify, manage and refer people with mental disorders as appropriate. Furthermore, in this context, the role of specialized mental health professionals needs to be expanded to encompass supervision and support of general health workers in providing mental health interventions [65].

Many health professionals are capable of task-sharing, as agreed upon by the member states. One example is professionals within physiotherapy, where a physiotherapeutic core task is to keep people active and to promote quality of life through treatment, prevention and health promotion [66]. Future physiotherapists are expected and able, in line with the Comprehensive Mental Health action plan, to work more innovatively within the field of health promotion [67]. The profession is constantly evolving and aims to ensure the best possible quality of life for the individual [66], encompassing physical, psychological, emotional, and social well-being through approaches that include health promotion, prevention, treatment, and rehabilitation [68]. Thus, as an example, and in support of task-sharing as a future route for the healthcare system to promote mental health, physiotherapy embraces a holistic perspective and is based on a biopsychosocial understanding [59], viewing mind and body as inseparable [28]. Among other healthcare professions, physiotherapy employs non-pharmacological methods to help individuals develop health literacy, enabling them to maintain and promote their own health and manage everyday activities [69].

### 4.1. Limitations and Strengths

This case study consists of only five participants, which limits the generalizability and calls attention to the need for further research exploring the experiences of winter bathing among people with ADHD. Nevertheless, our findings are important because they meet the need for further research to gain deeper insight into complementary non-pharmacological interventions and treatment options that may support mental well-being among people with ADHD and may have potential as adjuncts to pharmacological interventions either as supplements to medication or as stand-alone interventions [4,5,10,11]. According to data saturation, thematic saturation was reached with a minimum of four participants, and several were reached with all five participants.

We did not investigate directly in the present study how the participants coped in the summer months. However, some participants described supplementing winter bathing with other forms of cold-water exposure, such as streams or cold-water tubs. These may serve as alternatives to the sea, although seasonal patterns were not specifically examined.

The recruitment strategy relied on winter bathing clubs and social media, which inherently select individuals who already enjoy or have successfully adopted this practice. Therefore, individuals with ADHD who found cold water exposure intolerable or ineffective were not represented in this study.

The validity was strengthened by our fully participating in the cold water together with the participants. Additionally, by selecting the theoretical perspectives *after* the thematic analysis of the empirical data, we protected the empirical data from being overruled by theory.

The source triangulation between our findings and the descriptions from K, B and the HICE participants strengthened the confirmability of our results, suggesting relevant mechanisms for how winter bathing may enhance mental well-being for people with ADHD. It would have strengthened the validity and clinical relevance of the findings further to include and integrate the professionals’ clinical observations even more systematically in the discussion.

### 4.2. Perspectives

In psychiatry, individuals with ADHD, anxiety, and depression are often treated with medication and psychotherapy, but methods such as BBAT, music therapy, and physical activity are also used to promote calmness, joy, and bodily awareness [70,71]. Supporting emotional regulation and assigning the body an active role are particularly important in psychiatry, where many patients experience racing thoughts, restlessness, and stress. Our findings indicate that winter bathing as an activity may enhance calmness, focus, and well-being through cold exposure, nature, and bodily experiences, and thus potentially could be applied as a supplement to the existing treatment regimen already offered.

Through task-sharing, general health professionals could thus play an important role in facilitating or mediating winter bathing, given that future research establishes an evidence base for the beneficial effects of winter bathing on the mental well-being of individuals with ADHD. In this case, winter bathing could potentially be recommended as a supplementary intervention through Health Authority agencies and ADHD associations. This could also provide winter bathing clubs with a professional and health-related basis for allocating specific membership slots to targeted groups, to which physiotherapists or other health professionals could refer. Initiatives such as HICE already exist, in which winter bathing is offered to individuals with a neurodevelopmental condition. Here, physiotherapists and general health professionals could act as a link by referring relevant citizens to such initiatives as part of a health-promoting effort.

In the Danish public school system, there are several types of special classes that include children and young people with ADHD. For instance, Beder School in Aarhus Municipality actively works with special classes to meet the needs of pupils with special needs [72]. In addition, Gørvad Efterskole is a special boarding school aimed particularly at young people with ADHD [73]. It could be of interest to explore whether winter bathing could be implemented as part of the curriculum in special classes, for example, as physical education or as an activity at the boarding school, as part of strategies for pupils managing their ADHD. This would require further investigation into the experience of winter bathing among children with ADHD.

We hope that our study helps clarify how physiotherapists and other health professionals—through their expertise in the body, movement, and motivation—can play a valuable role in guiding institutions and agencies, as well as in supporting individuals with ADHD in engaging in activities that promote physical health and mental well-being. At the same time, our findings highlight a clear need for further research in this area, so that health professionals can base their guidance and recommendations on robust evidence when considering activities such as winter bathing as a health-promoting practice or as a supplement to treatment. We also hope that our study will inspire future research into how winter bathing can be integrated into a holistic strategy for working with individuals with ADHD.

## 5. Conclusions

In conclusion, winter bathing contributed to an immediate and lasting peace of mind and relief from racing thoughts, and it increased concentration and focus. Winter bathing enhanced the participants’ body awareness and created a sense of presence and balance in the mind–body. The joy of winter bathing was persistent and profound, with a positive impact on mood, energy, and overall zest for life. Furthermore, winter bathing emerged as an important strategy for emotional regulation and coping with everyday life, and several of the participants experienced that the calmness and benefits from winter bathing were transferred to other situations. The activity was used as a supplement and, for some, as an alternative to medical treatment for ADHD, anxiety, and depression. Several reported a reduction in symptoms and a decreased need for medication.

Our study indicates that winter bathing may represent a meaningful non-pharmacological intervention to enhance mental well-being for individuals with ADHD. Winter bathing in combination with nature, cold exposure, and bodily presence created a sensory space in which most of the participants in this study experienced mental well-being. Our findings are important because they meet the need for research on complementary non-pharmacological interventions supporting mental well-being among people with ADHD. Furthermore, our study provides a basis and relevance for future, larger-scale studies that can examine both the short- and long-term experiences of winter bathing for individuals with ADHD. Further research will be valuable to explore how winter bathing could potentially be incorporated into a health-promoting intervention.

## Figures and Tables

**Figure A1:**
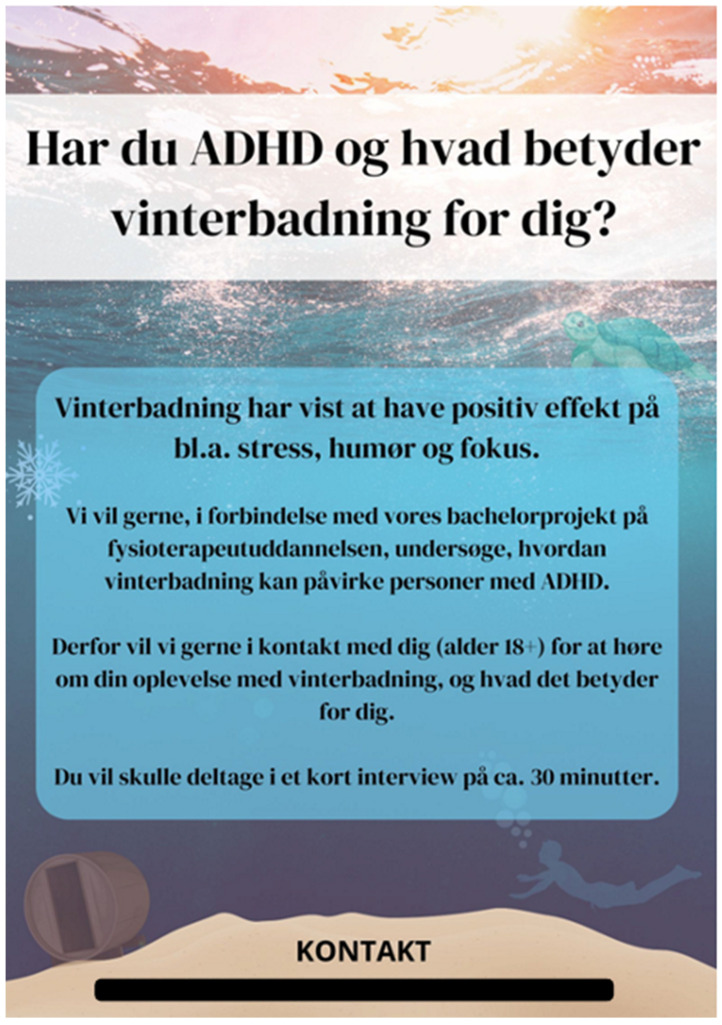

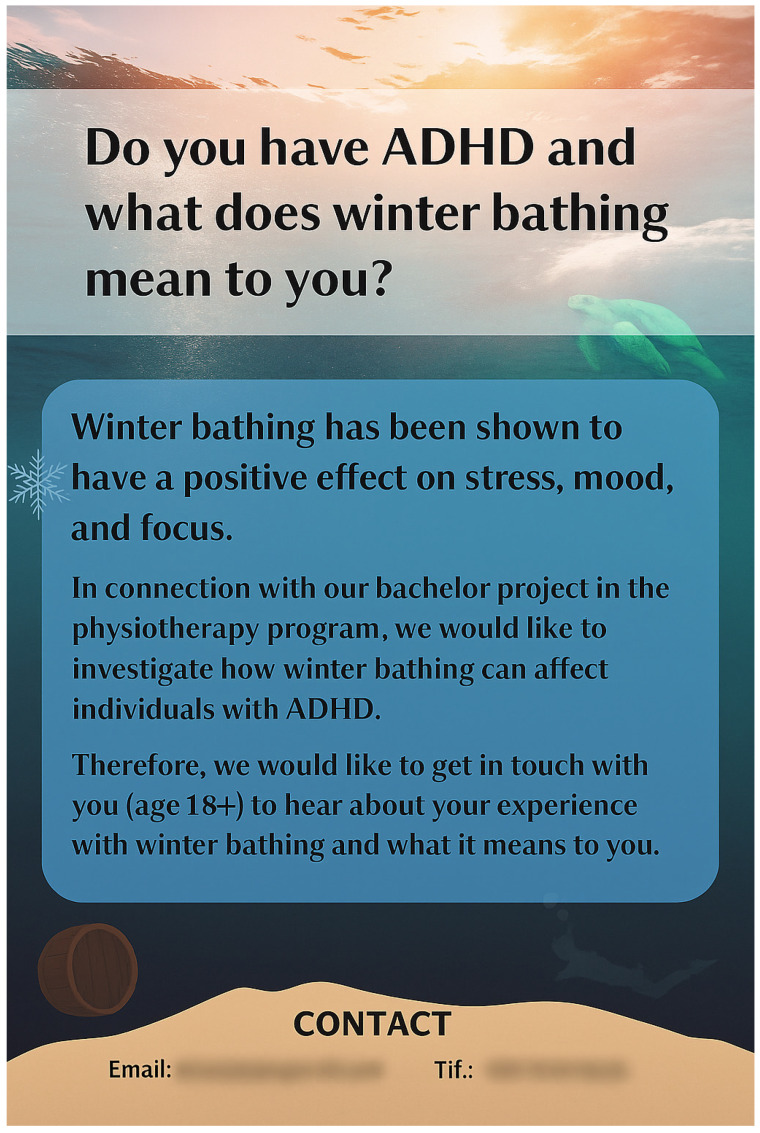
Facebook Poster (in Danish and in English).

## Data Availability

The original contributions presented in this study are included in this article; further inquiries can be directed to the corresponding author.

## Appendix A. Interview Guide for Participants

Research questions:

Hvordan oplever du at have ADHD?

Hvordan opleves vinterbadning?

Hvordan opleves effekten af vinterbadning på mennesker med ADHD?

Interview guide:

**Theme**	**Interview Questions**	**Follow-Up Questions**
Briefing + consent form	Purpose of the interview	- Introduce ourselves (role distribution) - Purpose of the interview - Time frame - Data anonymisation - Rights - May be included in an article - Recording (audio) - Consent form (provided)
ADHD	How do you experience having ADHD/ADD?	- How do you experience everyday life with ADHD? ○ Mentally ○ Physically ○ Relationships ○ Socially ○ Concentration ○ Planning ○ Impulsivity ○ Hyperactivity - How do you feel about having ADHD? - What do you experience as positive/challenging about having ADHD?
Winter bathing	How do you experience winter bathing?	- What does winter bathing mean to you? - Why do you winter bathe? - What made you start winter bathing? - Can you describe the first period after you started winter bathing? - How do you experience your body reacting in the water and afterwards? ○ Feelings/thoughts - How do you experience your mental well-being during winter bathing? - Have you experienced a particularly positive situation? - Have you experienced a particularly negative situation? - Do the surroundings where you winter bathe matter? (In what way?) - Are there social events? Significance?
Experience	How do you experience the effect of winter bathing on your ADHD?	- Can you describe your winter bathing journey? - Have you noticed emotional/physical/mental reactions to winter bathing that affect your ADHD? Which? - Can you feel a difference between the days you bathe and the days you do not bathe? - How does the effect influence your everyday life with ADHD? And for how long? How long does it take before you feel the need to winter bathe again? - Can you describe a situation where winter bathing affected your ADHD and made a particular impression on you? - How do you experience winter bathing’s impact on your ADHD in your relationships and everyday life? - Has winter bathing affected your use of ADHD medication?
Extra questions for perspective		- Could winter bathing be included as a supplementary activity to manage your ADHD? - Could winter bathing be included as a physiotherapeutic intervention as part of your treatment? - Do you have experience with other initiatives to manage your ADHD positively?
Information		- Age, education, occupation? - For how long have you been doing winter bathing? - How regularly do you bathe? - When were you diagnosed with ADHD? - Do you take medication for your ADHD? - Do you winter bathe alone or with others?
Debriefing		- Do you have anything else you would like to say? - Would you like us to send the project results by e-mail?

## Appendix B. Interview Guide for Psychologist and ADHD Mentor

ADHD

- What is your background and experience with individuals with ADHD?
- What are the most common challenges you see in individuals with ADHD in your practice?
- Do you notice an increase in adults being diagnosed with ADHD, and if so, why do you think that is?

Winter Bathing

- Do you have experience with clients who use winter bathing as a strategy to manage their ADHD?
- Based on your professional knowledge, what mechanisms could explain why winter bathing might potentially affect ADHD symptoms?
- Are there any risks/contraindications in recommending winter bathing to individuals with ADHD?
- How do you assess the effect of alternative methods such as mindfulness, exercise, or winter bathing compared to medical treatment?
- Do you notice a tendency for individuals with ADHD to seek alternative or supplementary treatments?
- Could you imagine winter bathing being included as part of a therapeutic intervention?
- Is there anything else we have not asked about that you consider relevant in relation to ADHD and possible interventions?

## References

[1] ADHD-Foreningen (2024). https://adhd.dk/wp-content/uploads/2024/11/ADHD-i-tal-2022-1.pdf.

[2] Sundhedsdatastyrelsen (2025). Medstat.dk. https://www.medstat.dk/.

[3] ADHD-foreningen Hvad er ADHD?. https://adhd.dk/om-adhd/hvad-er-adhd/.

[4] Sundhedsstyrelsen (2024). Kommissorium for Udarbejdelse af Nationale Kliniske Anbefalinger for Udredning og Behandling af ADHD Hos Voksne. https://www.sst.dk/-/media/Fagperson/NKA-NKR/ADHD-voksne-2024/Kommissorium-NKA-ADHD-voksne.ashx.

[5] Sundhedsstyrelsen (2025). Non-Farmakologisk Behandling af ADHD Hos Voksne: Nationale Kliniske Anbefalinger.

[6] Jennum P., Hastrup L.H., Ibsen R., Kjellberg J., Simonsen E. (2020). Welfare consequences for people diagnosed with attention deficit hyperactivity disorder (ADHD): A matched nationwide study in Denmark. Eur. Neuropsychopharmacol..

[7] Sundhedsstyrelsen (2017). Udredning og Behandling af ADHD Hos Voksne. https://www.sst.dk/-/media/Udgivelser/2015/NKR-ADHD-voksne/National-Klinisk-Retningslinje-ADHD-hos-voksne.ashx.

[8] Ørnberg J. (2022). Netdoktor.dk. Medicinsk Behandling af ADHD. https://netdoktor.dk/psykiatri/adhd/sygdomme/medicinsk-behandling-af-adhd/.

[9] Oliva F., Malandrone F., di Girolamo G., Mirabella S., Colombi N., Carletto S., Ostacoli L. (2021). The efficacy of mindfulness-based interventions in attention-deficit/hyperactivity disorder beyond core symptoms: A systematic review, meta-analysis, and meta-regression. J. Affect. Disord..

[10] Chaplin S. (2018). Attention deficit hyperactivity disorder: Diagnosis and management. Prog. Neurol. Psychiatry.

[11] Catalá-López F., Hutton B., Núñez-Beltrán A., Page M.J., Ridao M., Macías Saint-Gerons D., Catalá M.A., Tabarés-Seisdedos R., Moher D. (2017). The pharmacological and non-pharmacological treatment of attention deficit hyperactivity disorder in children and adolescents: A systematic review with network meta-analyses of randomised trials. PLoS ONE.

[12] Rasmussen S.K. (2019). Netdoktor.dk. Vinterbadning: Et Koldt Gys er Godt for de Fleste. https://netdoktor.dk/muskler-led-og-knogler/sport-og-motion/sygdomme/vinterbadning-et-koldt-gys-er-godt-for-de-fleste/.

[13] Knechtle B., Waśkiewicz Z., Sousa C.V., Hill L., Nikolaidis P.T. (2020). Cold Water Swimming—Benefits and Risks: A Narrative Review. Int. J. Environ. Res. Public Health.

[14] Søberg S., Löfgren J., Philipsen F.E., Pedersen B.K., Karstoft K., Scheele C. (2021). Altered brown fat thermoregulation and enhanced cold-induced thermogenesis in young, healthy, winter-swimming men. Cell Rep. Med..

[15] Reed E.L., Chapman C.L., Whittman E.K., Park T.E., Larson E.A., Kaiser B.W., Comrada L.N., Needham K.W., Halliwill J.R., Minson C.T. (2023). Cardiovascular and mood responses to an acute bout of cold water immersion. J. Therm. Biol..

[16] Østergaard E.B., Petersen A.A., van den Hengel L., Jensen A.M., Jensen N.B., Sparre P.W., Dahlgaard J. (2024). Winter Bathing in Denmark: A Qualitative Case Study on Winter Bathing’s Impact on Mental Health. Healthcare.

[17] Hjorth P., Sikjær M.G., Løkke A., Jørgensen A.M., Jørgensen N., Kaasgaard D.M., Rasmussen M.R.V. (2023). Cold water swimming as an add-on treatment for depression: A feasibility study. Nord. J. Psychiatry.

[18] Huttunen P., Kokko L., Ylijukuri V. (2004). Winter swimming improves general well-being. Int. J. Circumpolar Health.

[19] Herrnegger S. (2022). Incorporating Traditional Finnish Winter Swimming into Physiotherapy for Treating Chronic Pain: A Systematized Literature Review. Theseus. http://www.theseus.fi/handle/10024/794228.

[20] Li J., Hou J., Zhang L., Dou S., Yang L., Teng V., Zhang C., Sun H., Lu P., Guo Y. (2024). Exposure to blue space surroundings and depressive symptoms in young Chinese adults: The mediating role of sleep. Environ. Res..

[21] Espeland D., de Weerd L., Mercer J.B. (2022). Health effects of voluntary exposure to cold water—A continuing subject of debate. Int. J. Circumpolar Health.

[22] Gislason H. (2022). Trap.lex.dk. Havene i Danmark. https://trap.lex.dk/Havene_i_Danmark.

[23] Westh I.G. Antallet af Vinterbadere Tredoblet på ti år: “Det er en Trend, som er Kommet for at Blive.” DR. 3 December 2024. https://www.dr.dk/nyheder/regionale/fyn/antallet-af-vinterbadere-tredoblet-paa-ti-aar-det-er-en-trend-som-er-kommet.

[24] Den Danske Ordbog Ordnet.dk. Vinterbade. https://ordnet.dk/ddo/ordbog?query=vinterbade.

[25] World Health Organization Regional Office for Europe (2017). Urban Green Space Interventions and Health: A Review of Impacts and Effectiveness. https://cdn.who.int/media/docs/librariesprovider2/euro-health-topics/environment/urban-green-space-intervention.pdf?sfvrsn=a2e135f3_1&download=true.

[26] Madsen L.S., Poulsen D.V., Madsen L.S., Poulsen D.V., Sørensen T.B., Vibholm P., Mygind O., Maribo T. (2022). Natur og sundhed i praksis. Vi Skal Mere ud: Grønbog om Naturen som Ressource i Sundhedsindsatser.

[27] World Health Organization WHO.int. Promoting Well-Being. https://www.who.int/activities/promoting-well-being.

[28] Olesen J. (2002). Kroppens filosofi: Med baggrund i Maurice Merleau-Pontys forfatterskab. Kognit. Pædagogik.

[29] Merleau-Ponty M. (1996). Phenomenology of Perception.

[30] Antonovsky A. (2007). Helbredets Mysterium: At Tåle Stress og Forblive Rask.

[31] Bandura A. (1986). Social Foundations of Thought and Action: A Social Cognitive Theory.

[32] Ryan R.M., Deci E.L. (2022). Self-Determination Theory: Basic Psychological Needs in Motivation, Development, and Wellness.

[33] Deci E.L., Ryan R.M. (2000). The “What” and “Why” of Goal Pursuits: Human Needs and the Self-Determination of Behavior. Psychol. Inq..

[34] Deci E.L., Ryan R.M. (2002). Handbook of Self-Determination Research.

[35] Ravn I. (2021). Selvbestemmelsesteorien: Motivation, Psykologiske Behov og Sociale Kontekster.

[36] Wilson E.O. (1984). Biophilia.

[37] Wilson E.O., Kellert S.R. (1993). The Biophilia Hypothesis.

[38] Kaplan R., Kaplan S. (1989). The Experience of Nature: A Psychological Perspective.

[39] Tjørnhøj-Thomsen T., Reventlow S., Grønbæk M., Bruun Jensen B., Reventlow S. (2021). Kvalitative metoder. Forebyggende Sundhedsarbejde.

[40] Spradley J.P. (1980). Participant Observation.

[41] Dalland O. (2020). Metode og Oppgaveskriving.

[42] Martinsen B., Norlyk A. (2011). Tre kvalitative forskningstilgange. Sygeplejersken.

[43] Christensen L.K.T., Schwennesen N., Vallgårda S., Bornø Jensen A.M. (2021). Hermeneutik: Filosofi, analyse og metode. Forskningsmetoder i Folkesundhedsvidenskab.

[44] Kristensen D.B., Vallgårda S., Bornø Jensen A.M. (2021). Fænomenologi.Filosofi, metode og analytisk værktøj. Forskningsmetoder i Folkesundhedsvidenskab.

[45] Barth F. (1980). Sosialantropologien som Grunnvitenskap.

[46] Goffman E. (1989). On fieldwork. J. Contemp. Ethnogr..

[47] Malterud K. (2025). Kvalitative Forskningsmetoder for Medisin og Helsefag.

[48] Vinterbadeforeninger i Danmark Vinterbader.dk. Liste over Vinterbaderklubber i Danmark. https://www.vinterbader.com/Member/List.

[49] World Medical Association (2024). Wma.net. WMA Declaration of Helsinki—Ethical Principles for Medical Research Involving Human Participants. https://www.wma.net/policies-post/wma-declaration-of-helsinki/.

[50] Nationale Videnskabsetiske Komité, Videnskabsetiske Medicinske Komitéer (2024). Videnskabsetik.dk. Vejledning om det Informerede og Stedfortrædende Samtykke i Sundhedsvidenskabelige Forskningsprojekter. https://videnskabsetik.dk/vejledninger/vejledning-om-det-informerede-og-stedfortraedende-samtykke-i-sundhedsvidenskabelige-forskningsprojekter#heading28.

[51] Crocker A.F., Smith S.N. (2019). Person-first language: Are we practicing what we preach?. J. Multidiscip. Healthc..

[52] Malterud K. (2012). Systematic text condensation: A strategy for qualitative analysis. Scand. J. Public. Health.

[53] Bundgaard H., Overgaard Mogensen H., Bundgaard H., Rubow C., Overgaard Mogensen H., Ahl S.I. (2018). Analyse: Arbejdet med det etnografiske materiale. Antropologiske Projekter: En Grundbog.

[54] Wolcott H.F. (2001). The Art of Fieldwork.

[55] Poulsen I., Larsen S., Schou L., Høstrup H., Lyngsø E.E. (2009). VAKS—Vurdering af kvalitative studier. Sygeplejersken.

[56] Østergaard E.B., Nielsen B.W., Wiegaard L., Jørgensen M.M., Madsen H.N., Dahlgaard J.O. (2022). Den Glemte Krop: Når Berøring og Bevægelse Virker Positivt på Sindet til Fremme for Mental Sundhed. Tidsskrift.dk.

[57] Mitchell J.T., McIntyre E.M., English J.S., Dennis M.F., Beckham J.C., Kollins S.H. (2017). A Pilot Trial of Mindfulness Meditation Training for ADHD in Adulthood: Impact on Core Symptoms, Executive Functioning, and Emotion Dysregulation. J. Atten. Disord..

[58] Pagán A.F., Huizar Y.P., Short T.R., Gotcher Z., Schmidt A.T. (2023). Adult Attention-Deficit/Hyperactivity Disorder: A Narrative Review of Biological Mechanisms, Treatments, and Outcomes. Curr. Neurol. Neurosci. Rep..

[59] Hansen J., Bjørnlund I.B., Sjöberg N.E., Lund H. (2017). Sundheds-og sygdomsbegrebet i et historisk perspektiv. Basisbog i Fysioterapi.

[60] Djernis D., Lundsgaard C.M., Rønn-Smidt H., Dahlgaard J. (2023). Nature-Based Mindfulness: A Qualitative Study of the Experience of Support for Self-Regulation. Healthcare.

[61] Østergaard E.B., Sparre P.W., Dahlgaard J. (2024). Two-and-a-Half-Year Follow-Up Study with Freedom on Water through Stand-Up Paddling: Exploring Experiences in Blue Spaces and Their Long-Term Impact on Mental Well-Being. Healthcare.

[62] Merleau-Ponty M. (2009). Kroppens Fænomenologi.

[63] Bunkan B.H. (2014). Kropp, Respirasjon og Kroppsbilde: Teori og Helsefremmende Behandling.

[64] Østergaard E.B., Lund H., Bjørnlund I.B., Sjöberg N.E. (2013). At have, at skabe og at være en krop. Basisbog i Fysioterapi.

[65] World Health Organization (2021). Comprehensive Mental Health Action Plan 2013–2030. Geneva. https://www.who.int/publications/b/58680.

[66] Danske Fysioterapeuter Fysio.dk. Hvad kan Fysioterapeuter?. https://www.fysio.dk/om-os/hvem-er-vi/hvad-kan-fysioterapeuter.

[67] VIA University College (2023). Studieordning VIA Fysioterapeutuddannelsen. https://www.via.dk/uddannelser/fysioterapeut/studieordninger-fysioterapeut.

[68] Fraktion af Kliniske Undervisere i Fysioterapi (2011). Fkuf.dk. Hvad er fysioterapi?. https://www.fkuf.dk/historie/hvad-er-fysioterapi.

[69] Madsen M.H., Højgaard B. (2009). Health Literacy—Begrebet, Konsekvenser og Mulige Interventioner: Notat Udarbejdet for Sundhedsstyrelsen af Dansk Sundhedsinstitut.

[70] Region Midtjylland Psykiatrien i Region Midtjylland. Fysioterapi. n.d. https://www.auh.dk/afdelinger/psykiatriske-afdelinger/afdeling-for-depression-og-angst/fysioterapi/.

[71] (2023). Region Nordjylland, Psykiatrien. Psykiatri.rn.dk. Musikterapi. https://psykiatri.rn.dk/Diagnose-og-behandling/Musikterapi?utm_source.

[72] Aarhus Kommune Skoler med Specialklasser i Aarhus Kommune. https://aarhus.dk/media/iaahpahs/skoler-med-specialklasser-i-aarhus-kommune.pdf?format=noformat.

[73] Gødvad Efterskole Efterskole ADHD—En Specialefterskole i Jylland for Unge med ADHD og ADD. https://goed.dk/efterskole-adhd/.

